# How can macromolecular crowding inhibit biological reactions? The enhanced formation of DNA nanoparticles

**DOI:** 10.1038/srep22033

**Published:** 2016-02-23

**Authors:** Sen Hou, Piotr Trochimczyk, Lili Sun, Agnieszka Wisniewska, Tomasz Kalwarczyk, Xuzhu Zhang, Beata Wielgus-Kutrowska, Agnieszka Bzowska, Robert Holyst

**Affiliations:** 1 Institute of Physical Chemistry PAS, Kasprzaka 44/52, 01-224 Warsaw, Poland; 2 Division of Biophysics, Institute of Experimental Physics, Faculty of Physics, University of Warsaw, al. Zwirki i Wigury 93, 02–089 Warsaw, Poland

## Abstract

In contrast to the already known effect that macromolecular crowding usually promotes biological reactions, solutions of PEG 6k at high concentrations stop the cleavage of DNA by HindIII enzyme, due to the formation of DNA nanoparticles. We characterized the DNA nanoparticles and probed the prerequisites for their formation using multiple techniques such as fluorescence correlation spectroscopy, dynamic light scattering, fluorescence analytical ultracentrifugation etc. In >25% PEG 6k solution, macromolecular crowding promotes the formation of DNA nanoparticles with dimensions of several hundreds of nanometers. The formation of DNA nanoparticles is a fast and reversible process. Both plasmid DNA (2686 bp) and double-stranded/single-stranded DNA fragment (66bp/nt) can form nanoparticles. We attribute the enhanced nanoparticle formation to the depletion effect of macromolecular crowding. This study presents our idea to enhance the formation of DNA nanoparticles by macromolecular crowding, providing the first step towards a final solution to efficient gene therapy.

The formation of DNA nanoparticles is meaningful due to its impact on biological processes^1^,^2^. The formation of nanoparticles decreases DNA accessibility to enzymes and protects the DNA molecules from the damage caused by the enzymes. This protection is a prerequisite for dozens of biological processes. For example, exogenous DNA molecules have to form compact nanoparticles before they enter the nucleus in the gene transfection process^3^. Insufficient interaction of gene delivery materials with DNA is still a shortcoming for potential gene delivery materials which leads to low gene delivery efficiency^4^. Usually the interaction is promoted by the chemical modification of molecular structures^5^,^6^. However each of the methods is only valid for certain types of materials. In this study we bring to light a robust method to promote the formation of DNA nanoparticles by macromolecular crowding.

Macromolecular crowding is defined as the presence of high concentrations of macromolecules in a solution^7^. The study of macromolecular crowding is inspired by the fact that cytoplasm is composed of high concentrations of macromolecules such as DNA, RNA and proteins^7^. For example, the total concentration of RNA and protein inside *E. coli* cells is in the range of 300–400 g/L^8^. The macromolecular crowding conditions also occur in eukaryotic cells^9^. Macromolecules occupy a significant fraction of the volume (typically 20–30%) in cells. This steric exclusion makes biological reactions to have different characteristics to those that occur in a water buffer solution. The macromolecules occupy an overwhelmingly large fraction of the solution volume and reduce the solvent volume for other reaction molecules. As a result, the effective concentration of the reaction molecules increases and thus the reaction rate increases. This point of view has been supported by a large number of publications. Zimmerman *et al.* reported that macromolecular crowding increases the reaction rate of DNA ligase^10^. Sasaki *et al.* reported that macromolecular crowding enhances DNA cleavage by endonuclease DNase I but has little effect on exo III and exo I exonucleases^11^. In another study, Zimmerman *et al.* reported that macromolecular crowding enhances the binding of DNA polymerase to DNA^12^. Unfortunately, these results imply indirectly that macromolecular crowding should not cause the formation of DNA nanoparticles, because in DNA nanoparticles the reaction rate should decrease since the compact structure inhibits enzymes from approaching the DNA. Contrary to the numerous results, in this study we unveil the effect that certain macromolecular crowding triggers the formation of DNA nanoparticles.

The formation of DNA nanoparticles promoted by macromolecular crowding is indirectly supported by several early studies where the reaction rate of several biological reactions was decreased. Tan *et al.* reported that the gene expression rate in Poly(ethylene glycol) (PEG) 8k solutions undergoes at first an increase, then a decrease and finally comes to a complete halt with increase of PEG concentration^13^. We also found that the macromolecular crowding environment composed of PEG 6k solution effectively inhibits the cleavage of DNA by the HindIII restriction enzyme^14^. These studies indirectly imply that DNA probably forms compact nanoparticles that inhibit the enzyme from approaching the DNA. It is obvious that the effect does not apply to all types of macromolecules. Only a few macromolecules as far as we know, such as PEG 6k, are reported to be capable of inhibiting the biological reaction, i.e. promoting DNA nanoparticle formation. Therefore, we chose PEG 6k as the macromolecules in our study.

When two DNA molecules come close to each other in the presence of flexible polymers, the space between the DNA molecules is depleted of the polymer coils, when their relative distance is smaller than the polymer radius of gyration. This depletion of coils between the DNA molecules causes an imbalance in the osmotic pressure and leads to an effective attraction between the DNA fragments^14^. The attraction is strong when the size of the flexible polymer is comparable to the radius of the DNA cylinder^15^. This effect makes the formation of DNA nanoparticles possible by the appropriate choice of polymer molecules.

In this study based on the discovery of the unusual phenomenon that macromolecular crowding inhibits DNA cleavage, we combined multiple techniques such as fluorescence correlation spectroscopy (FCS), dynamic light scattering (DLS), fluorescence analytical ultracentrifugation assay (FAUC), etc to carry out a systematic study of the formation of DNA nanoparticles in PEG 6k solution. We report for the first time that macromolecular crowding promotes the formation of DNA nanoparticles with dimensions of hundreds of nanometers, in contrast to numerous previously reported results^12^,^16^. We characterized and analyzed the prerequisites for the formation of DNA nanoparticles.

## Result

### DNA cleavage inhibited by macromolecular crowding

Contrary to a large number of reports where biological reactions are enhanced by macromolecular crowding^11^,^12^, we show that a simple and typical biological reaction, the cleavage of DNA by restriction enzyme, is inhibited by macromolecular crowding. The cleavage of plasmid DNA is observed by electrophoresis mobility in agarose gels. Native plasmid DNA has a negatively super-coiled structure. Introducing single-stranded cleavage releases the topological pressure in the plasmid DNA and convert it into loose circular one. The cleavages in both strands in a close vicinity convert the DNA into a linear structure. The three types of DNA have different electrophoresis motilities (see the relation of DNA type to its electrophoresis mobility in the supporting information). The cleavage of DNA can be traced by the relative amount of the three DNA components shown in the electrophoresis gel.

**Figure 1:**
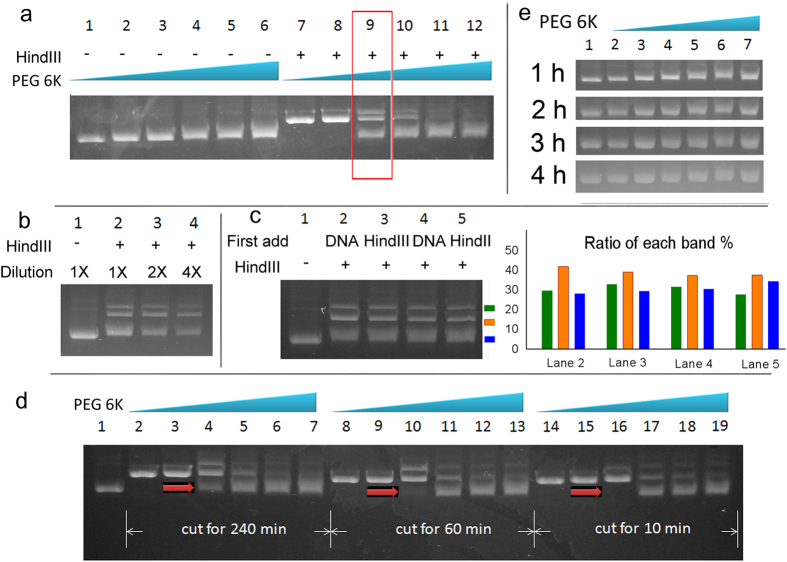
Inhibited DNA cleavage in macromolecular crowding environment detected by electrophoresis assay. (**a**) DNA cleavage by HindIII is inhibited in macromolecular crowding solution. The concentrations of PEG 6k in lanes 1–6 (and lanes 7–12) are 0%, 6.25%, 12.5%, 18.75%, 25% and 31.25% respectively. Samples in lanes 1–6 contain no HindIII enzyme. The concentration of DNA is the same in each lane. The concentration of HindIII enzyme is the same as in Lanes 7–12. (**b**) The extent of DNA cleavage in 12.5% PEG 6k solution shows no difference in the presence of different concentrations of DNA and HindIII enzyme. The concentrations of DNA and HindIII in lane 2 are the same as those in lane 9 in Fig. 1a . The concentrations of DNA and HindIII in lane 3 and 4 are 1/2 and 1/4 of those in lane 2. (**c**) Neither the reaction temperature nor the sample addition sequence affects the extent of cleavage. For lanes 2 and 4, we added DNA first, waited for 10 min and then added HindIII to initiate the cleavage process. For lanes 3 and 5, we added HindIII first. The concentration of PEG 6k solution is 12.5%. The samples in lanes 2–4 were the same as in lane 9 in Fig. 1a. The reaction temperature for the samples in lanes 2 and 3 was 23 °C and for Lanes 4 and 5 was 37 °C. Lane 1 shows pUC19 DNA without cleavage. (**d**) Three sets of cleavage experiments were initiated simultaneously with the same components as in Fig. 1a. We added 2 μl of loading buffer to the cleavage mixture to stop the reaction after 10 min, 60 min and 240 min respectively. Then the samples were loaded simultaneously for electrophoresis assay. (**e**) The DNA prepared in the macromolecular crowding environment did not show retardation in the electrophoresis assay (without HindIII). The components in lanes 1–6 is the same as in Fig. 1a lanes 1–6. In order to make a clear comparison between the mobility of the samples in lane 1 and lane 6, we loaded the same sample as lane 1 into lane 7.

The cleavage of DNA by the HindIII restriction enzyme is gradually inhibited as the PEG 6k concentration increases. Obvious inhibition can be observed in the presence of 12.5% PEG 6k. Further inhibition can be observed in the presence of 25% PEG 6k (Fig. 1a). We compared the ratio of different types of DNA in 12.5% PEG 6k to check the extend of the cleavage. In 12.5% PEG 6k three types of DNA components coexist after the cleavage of DNA (Fig. 1a). Their ratio is not affected after we decrease the concentration of DNA and the Hind III enzymes (Fig. 1b). Since the necessary cutting time for the complete cut should be within 10 min (Fig. 1d), the cutting time (1 h) is enough for a complete DNA cleavage process.

In macromolecular crowding environment, no proteins are pushed to the DNA surface to block the enzyme’s target site on the DNA, as shown by the FCS experiment on BSA attachment to DNA (Fig. S9). We explained the inhibition of DNA cleavage as the formation of DNA nanoparticles hampering the approach of HindIII enzymes to their target site on DNA. In the presence of 12.5% PEG 6k, the DNA forms DNA nanoparticles with semi-compact structures. HindIII enzymes can only cut the edge of the DNA nanoparticles with loose structures but cannot approach the central part. As a result a fixed proportion of DNA is cut in a certain concentration of PEG 6k.

We added either DNA or HindIII to the mixture, waited for 10 min and finally added the other buffer components for cleavage to initiate the reaction. Figure 1c shows that no visible difference was observed in the cleavage extent between the two samples at either room temperature (23 °C) or the enzyme cleavage temperature (37 °C). If the DNA molecules need considerable time to prepare their structure, when we incubated the DNA for 10 min in PEG 6k solution before adding HindIII to change their structure, this DNA might have been better protected and less cut. However such a result was never observed. Therefore the formation of DNA nanoparticles is a rapid process.

Usually the formation of DNA nanoparticles or DNA aggregates can be detected by the retarded DNA mobility in the electrophoresis assay^17^. However the mobility of DNA is just slightly reduced in the enzyme buffer system without HindIII enzyme (Fig. 1a). (We explained the slight decrease in the electrophoresis ability of DNA bands as being due to the viscous PEG 6k solution reducing the mobility of DNA molecules at the beginning of the electrophoresis assay. See the viscosity of PEG 6k solution in the supporting information.) Figure 1e shows that the DNA samples prepared with different concentrations of PEG 6k solutions moved with the same mobility 1 h after entering the gel. The result indicates that the structures of DNA are the same. During the electrophoresis assay, the solution with macromolecular crowding is gradually replaced by the electrophoresis buffer (PEG 6k should be immobile in the electrophoresis assay since it is electrically neutral). As a result the DNA nanoparticles disassemble in the agarose gel. The disassembly of DNA nanoparticles suggests that they are unstable when removed from the macromolecular crowding environment.

### The formation of DNA nanoparticles with plasmid DNA

The DNA nanoparticles formed with plasmid DNA as shown in the electrophoresis assay were confirmed by DLS. We used the cleavage buffer system as the DNA solution to mimic the experimental conditions in the electrophoresis assay. According to Eqs 3 and 5, the size of the particles is proportional to the characteristic decay time of the DLS autocorrelation signal. The characteristic time can be roughly estimated as the position of the turning point. Therefore comparing the shape of DLS autocorrelation curves we can see the formation of DNA nanoparticles.

**Figure 2:**
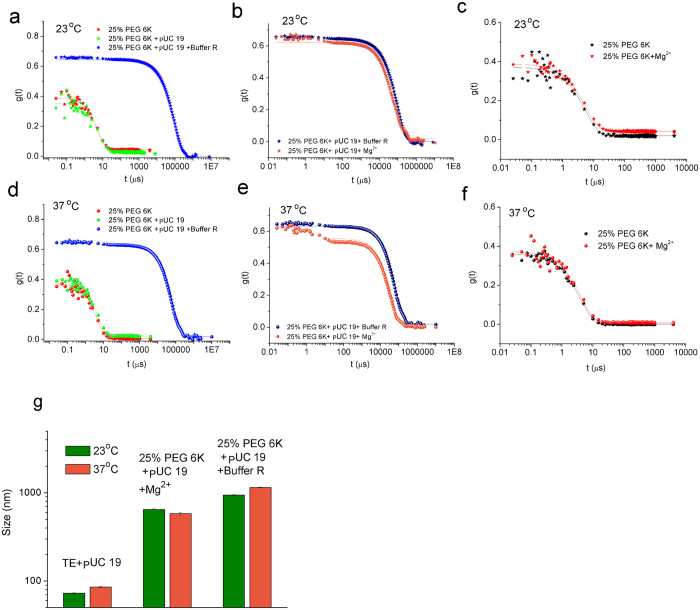
Formation of DNA nanoparticles with plasmid DNA pUC 19 studied by DLS. The autocorrelation curves in (**a**,**d**) show that the addition of Buffer R into the PEG 6k solution induces large particles. However, PEG 6k alone cannot cause the phenomenon. The autocorrelation curves in (**b**,**e**) show that Mg^2+^, one component in Buffer R, has the same function as Buffer R to induce the formation of large particles. The autocorrelation curves in (**e**,**f**) show that no such large particles can be found in PEG 6k solution even after adding Mg^2+^, where no DNA is present. Therefore the particles should be composed of DNA. (**g**) shows the shift of size of DNA before and after the appearance of large particles.

The formation of DNA nanoparticles requires the presence of both macromolecular crowding and Buffer R (Fig. 2a,d). Macromolecular crowding alone cannot cause the formation of large particles (Fig. 2a,d). The same concentration of Mg^2+^ as in Buffer R causes a similar effect (Fig. 2b,e). Also, nanoparticles are not formed if DNA is absent (Fig. 2c,f), indicating that the nanoparticles are composed of DNA. When the DNA forms nanoparticles, their size increases from ca. 70 nm (free state in water buffer) to hundreds of nanometers (in the PEG 6k solution) (Fig. 2g). (See also the size of particles in PEG 6k solution without DNA in the supporting information Fig. S10.) Assuming the hydrodynamic radius of DNA does not collapse due to their compacting inside the DNA nanoparticles and assuming the DNA nanoparticles are composed solely of DNA, there should be at least 1000 plasmid DNA inside one DNA nanoparticle on average.

The DLS signal shows the formation of large DNA nanoparticles in the macromolecular crowding environment, but the formation of DNA nanoparticles cannot be observed by their mobility in the electrophoresis assay (Fig. S11). Electrophoresis assay is a well-accepted method for checking the formation of DNA nanoparticles. It fails to observe DNA nanoparticles, which are unstable when outside the macromolecular crowding environment. Also, the morphology of DNA nanoparticles cannot be observed by microscopic methods such as TEM and AFM. The staining process in TEM changes macromolecular crowding environment and thus affects the DNA nanoparticles. A large amount of macromolecules significantly increases the background noise in AFM observation. Real-time techniques should be used to study the formation of DNA nanoparticles.

### The formation of DNA nanoparticles with fragment DNA

**Figure 3:**
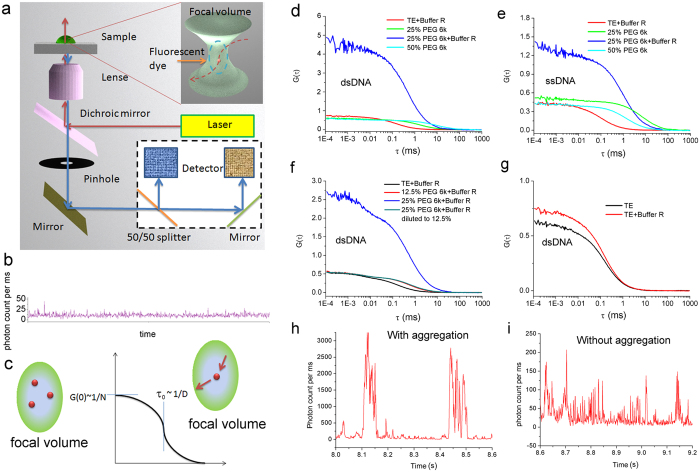
Formation of DNA nanoparticles with DNA fragments studied by FCS. 66 bp dsDNA and 66 nt ssDNA labeled with ATTO 488 dyes were used. (**a**) The FCS setup. A laser beam (488 nm) passes through a water-immersed object and focuses into a focal volume of a scale of hundreds of nanometers. The emitted fluorescence signal is collected, focused onto a pinhole by a lens, split into two and detected by avalanche photodiodes. (**b**) A typical FCS original signal collected by avalanche photodiodes. The fluorescence intensity reflects the number of photons collected by the detector. (**c**) A typical autocorrelation (cross-correlation) curve for a single-component sample solution. The amplitude of the FCS curves is inversely proportional to the number of fluorescence components in the focal volume. The characteristic diffusion time τ_0_ is inversely proportional to the diffusion coefficient of the tracers. From the FCS autocorrelation curves, we can trace the changes of the number and the size of labeled DNA molecules. In the presence of either 25% PEG 6K (**d**) or Buffer R (**g**), the concentration of DNA does not change obviously. However in the presence of both 25% PEG 6k and Buffer R, the amplitude and characteristic diffusion time of FCS autocorrelation curves decrease. Occasionally the FCS autocorrelation curves cannot be constructed based on the original fluorescence signal (**h**). The result indicates the formation of DNA nanoparticles. (**e**) The influence of PEG 6k and Buffer R on ssDNA is similar to dsDNA. Aggregation of dsDNA is a highly reversible process as a function of concentration of PEG 6k (**f**). The dsDNA sample in 12.5% PEG 6k has the same FCS autocorrelation curves as in PEG 6k which is diluted from 25% to 12.5%. (**h**,**i**) show FCS original signals with and without huge peaks.

Not only DNA plasmids but also dsDNA and ssDNA fragments are capable of forming DNA nanoparticles by macromolecular crowding. We labeled 66 bp/nt DNA fragments with fluorescent dyes and observed the DNA molecules in PEG 6k solution by FCS. We focused a laser beam into the focal volume and shone the sample inside. The original signal collected by the FCS detectors was proportional to the intensity of fluorescence in the focal volume. The original signal was autocorrelated to build the FCS autocorrelation curves (Fig. 3a). We obtained the information about the labeled DNA by fitting the FCS autocorrelation curve (Fig. 3b). When the geometry of the laser focal volume is constant (see the calibration of FCS system in supporting information), the amplitude of the FCS signal is proportional to the amount of labeled DNA in the focal volume, and the characteristic diffusion time is inversely proportional to the diffusion coefficient of the labeled DNA (Fig. 3c). Consequently if several DNA molecules become one nanoparticle, we should see from the FCS autocorrelation curves that the number of shining particles decreases and they diffuse more slowly.

In the presence of both Buffer R and 25% PEG 6k in the DNA solution, a significant shift in the FCS autocorrelation curves occurs (Fig. 3d). Meanwhile huge signal peaks are present in the original FCS signals (Fig. 3h). Occasionally the FCS autocorrelation curves cannot be built when the huge peaks are involved in the autocorrelation process. These results indicate the formation of large particles. No obvious changes are observed in the FCS autocorrelation curves for either dsDNA or ssDNA after the addition of PEG 6k to a final concentration of up to 50% (Fig. 3d,e). This result indicates that neither PEG 6k alone nor Buffer R can cause the formation of DNA nanoparticles. Assuming that each huge peak results from a single DNA nanoparticle and that the height of peaks is proportional to the number of DNA molecules inside, we roughly estimate that there are at least 60 DNA molecules inside one nanoparticle. In fact the fluorescent intensity of dye will decrease in nanoparticles (see the fluorescence spectra measurement section). The actual number of DNA molecules inside one nanoparticle should be even larger.

FCS data also show that the formation of DNA nanoparticles is a reversible process regulated by the concentration of macromolecular crowding environment. The DNA nanoparticles exist in 25% PEG 6k solution but not in 12.5% PEG 6k solution (Fig. 3f). When we increased the concentration of PEG 6k from 12.5% to 25%, DNA nanoparticles appear in the solution. We diluted the concentration of PEG 6k from 25% to 12.5% and maintained the same concentration of the other components. The DNA nanoparticles disappeared after dilution. The FCS autocorrelation curve for the 12.5% PEG 6k sample is the same as for the PEG 6k sample diluted from 25% to 12.5%.

We calculated the proportion of DNA fragments involved in the formation of DNA nanoparticles. We obtained information about the free DNA from the FCS autocorrelation curves which were constructed with the FCS original signals without the huge peaks (shown as the autocorrelation curves in Fig. 3d,e for the sample marked with “25% PEG 6k + Buffer R”). Both dsDNA and ssDNA can form DNA nanoparticles in the presence of both 25% PEG 6k and Buffer R. The proportion of free DNA in 25% PEG 6k solution in the presence of Buffer R was different for dsDNA and ssDNA. On average ~20% dsDNA and ~35% ssDNA were free in PEG 6k solution with Buffer R. (We obtained the number of fluorescent particles by comparing the amplitude of the corresponding FCS autocorrelation curves.) The result indicates that dsDNA is more inclined to form DNA nanoparticles than ssDNA in macromolecular crowding environment.

We did not observe the formation of DNA nanoparticles in 12.5% PEG 6k solution in FCS experiments, because we used short DNA fragments (66 bp) of very small concentration (in nM range i.e. ca. 0.04 mg/l). These DNA fragments also form nanoparticles in 25% PEG 6k. The concentration of DNA in the FCS experiment is limited by the FCS technique to a few nM. The nanoparticles formed in this experiment were detected by a sudden increase of light intensity emitted in the confocal volume, indicating the formation of DNA nanoparticles. In the electrophoresis assay, we used plasmid DNA of a much higher concentration of 50 mg/l.

**Figure 4:**
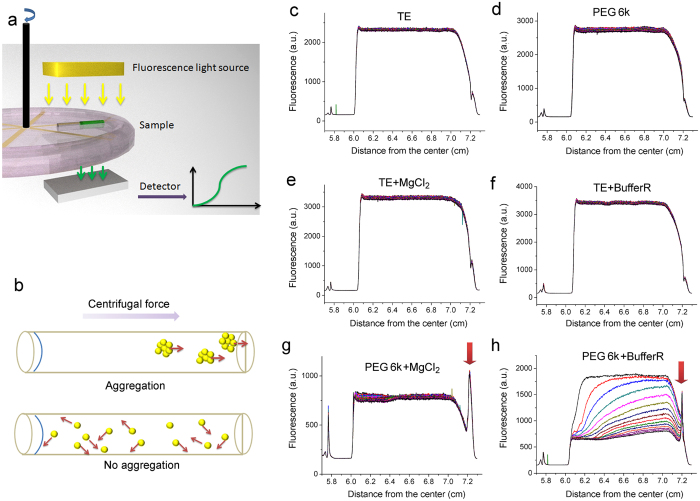
Formation of DNA nanoparticles with DNA fragment studied by FAUC. (**a**) Fluorescence analytical ultracentrifugation assay set up. We labeled the 66 bp dsDNA fragments with FAM dye to check the sedimentation of DNA under different conditions by FAUC. In order to capture the sedimentation process of large nanoparticles with an extremely high sedimentation coefficient, the experiments were performed at 5000 rpm for 80 min. (**b**) When DNA forms large aggregates they sediment to the bottom quickly. The sedimentation coefficient is proportional to the mass of the DNA aggregates. No obvious sedimentation of dsDNA was observed in TE buffer (**c**), in 25% PEG 6K solution (**d**), in TE Buffer solution supplemented with 10 mM Mg^2+^ (**e**), or in TE Buffer solution supplemented with Buffer R (**f**). The accumulation of DNA was observed on the bottom of the sample holder in 25% PEG 6k solution supplemented with 10 mM Mg^2+^ (**g**) and in 25% PEG 6k solution supplemented with Buffer R (**h**). Obvious sedimentation of dsDNA was observed in 25% PEG 6k solution supplemented with Buffer R (**g**). The time span between neighboring curves was 6 min. The samples for FAUC were located at 6.0 ~ 7.2 cm from the rotation center.

We further confirmed the FCS result by FAUC. We labeled 66bp dsDNA fragments with FAM dye and observed their behavior in the field of centrifugal force. The sedimentation coefficient of dsDNA is 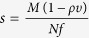, where *N* is Avogadro’s number, *M* is the molar weight of the solute, *f* is the frictional coefficient, υ is the partial specific volume of the solute and *ρ* is the density of the solvent. In PEG solution, *υ* of DNA is usually between 0.55–0.59 cm^3^/g. The densities *ρ* of TE Buffer and PEG solution with or without Buffer R or Mg^2+^ are quite similar to each other (see the measurement of density of the solutions in the supporting information). Assuming that the frictional coefficient *f* of PEG 6k solution is constant, the sedimentation coefficient *s* is proportional to the mass of the sedimentation object. When DNA forms large nanoparticles with a larger mass, they will sediment with a higher velocity (Fig. 4b). No PEG 6k sedimentation occurred under the experimental conditions (the proof showing that no PEG 6k sedimentation occurs under 50,000 rpm, c.a. 200,000 g, for 180 min can be found in the supporting information), indicating that the solution conditions kept constant during FAUC measurement.

In the field of centrifugal force at 5000 rpm (c.a. 2000 g) no visible sedimentation of dsDNA is observed in 80 min in pure TE Buffer (Fig. 4c). Addition of neither Buffer R, Mg^2+^ nor 25% PEG 6k alone causes obvious sedimentation of dsDNA (Fig. 4d–f). Addition of PEG 6k and Buffer R together causes obvious sedimentation of dsDNA (Fig. 4h). These DNA molecules accumulate at the bottom of the holder and form a signal peak (shown by the red arrow in Fig. 4h). The result indicates the formation of large DNA nanoparticles. Adding the same concentration of Mg^2+^ as in Buffer R causes a similar signal peak on the bottom (Fig. 4g). These results agree well with the DLS and FCS measurements. However, in this case we failed to see the sedimentation process. For each analytical ultracentrifugation assay, the samples had to be centrifuged for several minutes (5,000 rpm) to calibrate the system with a similar speed as used in the FAUC assay. It seems the sedimentation of DNA in 25% PEG 6k solution with Mg^2+^ has been completed in the calibration process.

After the redistribution of dsDNA in the field of centrifugal force, the fluorescence signal always remained at a level of about 700 a.u. (Fig. 4g,h). This fluorescence signal was from the free dsDNA, which could not efficiently sediment in the field of centrifugal force used in the experiment. The observation of free dsDNA in FAUC was consistent with the FCS result.

### The formation of DNA nanoparticles influences the fluorescence of labeled DNA

**Figure 5:**
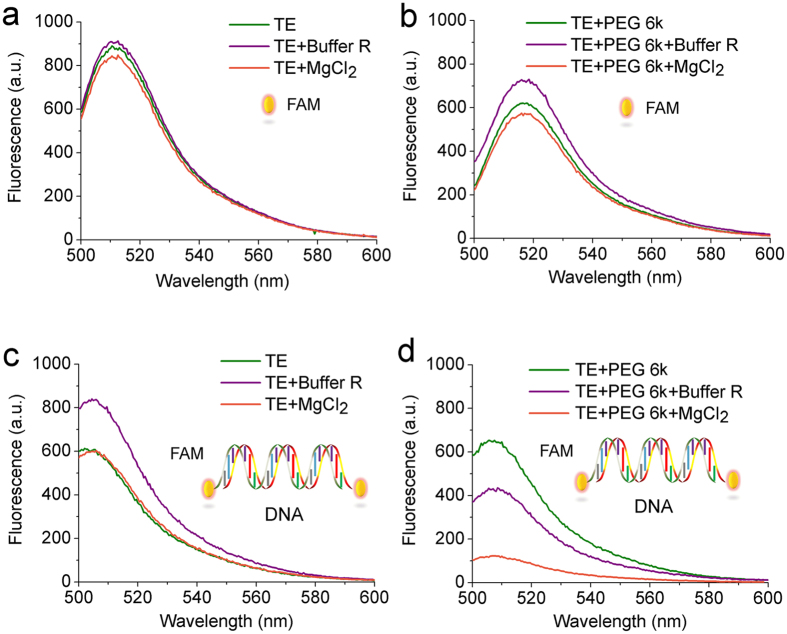
The formation of DNA nanoparticles influences the fluorescence of dyes used to label DNA. Fluorescence spectra for λ_exc_ = 488 nm of (**a**) free FAM dye in TE Buffer, (**b**) free FAM dye in 25% PEG 6k solution, (**c**) dsDNA labeled with FAM in TE Buffer , and (**d**) dsDNA labeled with FAM in 25% PEG 6k solution. Addition of Buffer R or Mg^2+^ does not have an obvious effect on the fluorescence intensity of free FAM dye or FAM-DNA in TE Buffer. Addition of Buffer R (violet line) or Mg^2+^ (orange line) does not have an obvious effect on the fluorescence intensity in the case of free FAM dye in PEG 6k solution. A significant decrease of the fluorescence intensity is observed when addition of either enzyme buffer conditions or Mg^2+^ for FAM-DNA in PEG 6K solution. The aggregation of DNA is the cooperative result of both PEG 6k and Buffer R or Mg^2+^.

The formation of DNA nanoparticles causes a shift in the structure of DNA from the free state to compact particles. It affects the fluorescence spectra of the dye with which the DNA is labeled. The addition of PEG 6k and Buffer R (or Mg^2+^) together into TE Buffer caused a significant decrease in the intensity of fluorescence of the DNA-FAM (compare Fig. 5c,d). Neither 25% PEG 6k, Buffer R nor Mg^2+^ alone caused a significant shift in the fluorescence spectra of DNA-FAM. The change of fluorescence intensity agrees with the formation of DNA nanoparticles. The result can be explained as the quenching of FAM fluorescence in the particular conditions inside DNA nanoparticles. The result confirms the conclusion that the formation of DNA nanoparticles requires both macromolecular crowding and solution context. The result is consistent with that obtained from DLS, FCS and FAUC measurements.

What should be noted is that the fluorescence intensity of free FAM does not undergo any change in the presence of either PEG 6k or Buffer R (Fig. 5a,b). The result suggests that macromolecular crowding does not directly affect the fluorescence of the dye.

## Discussion

Before our study, numerous reports on the effect of macromolecular crowding depicted the beautiful picture that macromolecular crowding almost always promotes biological reactions. Macromolecular crowding enhances the removal of antigen from the cell surfaces^18^, allows some reactions to occur which are impossible under conventional conditions^10^, increases the gene expression rate^13^,^19^, etc. All these can be attributed to the excluded volume effect: The macromolecules occupy a large volume in the solution and reduce the solvent volume for other macromolecules. Consequently the excluded volume effect increases the effective concentration of the reaction molecules and increases the reaction rate. Although our study shows that macromolecular crowding inhibits DNA cleavage, challenging the universal role of macromolecular crowding, yet we do not exclude the existence of the excluded volume effect. This excluded volume effect still plays a role in accelerating the cleavage of DNA. Now the question is: Why does macromolecular crowding play a distinct role in our study? After excluding the influence of viscosity (see a discussion about the role of viscosity in the supporting information), the answer lies in the much more powerful effect of macromolecular crowding, namely the depletion effect.

Although the detailed mechanism of how the depletion effect promotes the formation of DNA nanoparticles in macromolecular crowding environment is not clear, we can find some clues in the literature. The depletion effect of macromolecular crowding has been mentioned in the DNA condensation field under the name of DNA ψ-condensation. The researchers found that the structure of DNA molecules underwent collapse in the presence of PEG, as observed by circular dichroism (CD) spectra^20^. Fluorescence microscopy observation also revealed that giant T4DNA become shorter in the presence of PEG^21^. The depletion effect arises from the conformational entropy of semiflexible polymer coils. The center of mass of the polymer cannot get closer to the DNA than a characteristic distance. When the DNA molecules are separated by a distance less than this characteristic distance, the polymer molecule cannot enter the space between the two DNA molecules. This depletion zone between the DNA molecules causes an imbalance of osmotic pressure, which attracts the DNA molecules to each other^15^.

The depletion effect is determined by the size of the polymer coil and the radius of the DNA cylinder. A strong depletion effect requires the size of polymers to be close to that of the radius of the DNA cylinder^15^. This viewpoint is supported by a previous study concerning the influence of macromolecular crowding on gene expression^13^. Tan *et al.* studied the influence of Dex-small and Dex-big solution on gene expression. They reported that 10% Dex-small (M_w_ = 6k) could significantly inhibit the gene expression; while 10% Dex-big (M_w_ = 2M) could not. The radius of DNA cylinder is 1 nm, which is comparable to the size of 10% Dex-small (~2 nm). Consequently, the depletion effect was strong enough to cause DNA to form nanoparticles and to inhibit gene expression. On the other hand, the size of Dex-big is much larger than the radius of DNA, so the gene expression was not influenced. Actually, we also observed a similar inhibition of gene expression by PEG 6k in our lab (data not yet published), in agreement with Tan’s results. Wenner *et al.* reported that Ficoll 70 (5.5 nm) did not inhibit the cleavage of DNA by DNase EcoR V^16^. This result also confirms that the size of polymer matters in the depletion effect and the formation of DNA nanoparticles.

**Figure 6:**
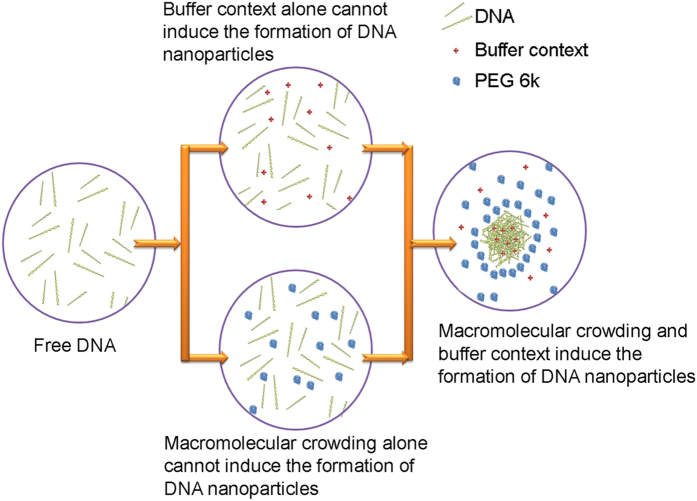
A model describing the prerequisites for the formation of DNA nanoparticles by macromolecular crowding. Due to the electrostatic repulsive force, DNA molecules are separated from each other in normal buffer solution. The buffer context, which can neutralize the charge of the DNA, screens the repulsive forces between them. Macromolecular crowding, which causes a strong depletion effect, exerts an attractive force between the DNA molecules. With the presence of the two factors, the attractive force between the DNA molecules overcomes the repelling force and DNA form nanoparticles. However neither of them can do it alone.

The depletion attraction force does not lead to the undoubted formation of DNA nanoparticles. DNA molecules tend to repel each other in solution because of their strong negative charge. PEG 6k causes an attracting force caused by a depletion effect which counterbalances the repelling force caused by electrostatic charges. When the attracting depletion force is weaker than the electrostatic repulsion force, macromolecular crowding is not able to induce the formation of DNA nanoparticles. Buffer R, especially Mg^2+^ can weakly interact with DNA. It is reported that Mg^2+^ can attract DNA onto the negatively charge mica surface by neutralizing the charge of DNA^22^. Mg^2+^ is also able to induce a certain level of DNA condensation^23^. However, this interaction between Mg^2+^ (or Buffer R) and DNA is still too weak to cause the formation of DNA nanoparticles to inhibit enzyme activity. However, the combination of macromolecular crowding and weak interacting factors such as Mg^2+^ together overwhelms the electrostatic repulsion force between DNA molecules. As a result the DNA molecules form nanoparticles (Fig. 6).

The DNA nanoparticles formed in the macromolecular crowding environment have similar functions to the DNA nanoparticles in the gene transfection system. These DNA nanoparticles are tight enough to stop enzymic cleavage, which meets the requirement for the DNA nanoparticles in the initial step of the gene transfection process^3^. These DNA nanoparticles are fragile when outside the macromolecular crowding environment, which also meets the requirement that DNA should be able to be released from the gene delivery complexes later in the gene transfection process^3^. Successful gene transfection involves more elements than the formation of DNA nanoparticles, i.e. the improved formation of DNA nanoparticles may not always bring about good gene transfection efficiency. Also, the influence of macromolecular crowding on gene delivery efficiency may differ from one kind of gene delivery material to another. More effort is still required to succeed in the battle for a better future in gene therapy.

To sum up, our study shatters the beautiful picture that macromolecular crowding always improves biological reactions—we have shown an inhibition of DNA cleavage by macromolecular crowding and unveiled the improved formation of DNA nanoparticles. The formation of DNA nanoparticles, which is impossible in a water buffer solution, is brought about by macromolecular crowding. The formation and disassembly of DNA nanoparticles is a fast and reversible process, regulated by the concentration of the macromolecules. Both plasmid DNA and double-stranded/single-stranded DNA fragments (66bp/nt) are capable of forming nanoparticles in macromolecular crowding environment. Both macromolecular crowding and buffer conditions are indispensable for the formation of DNA nanoparticles. Apart from the already known depleted volume effect that is responsible for enhanced biological reactions by macromolecular crowding, we have brought to light a size dependent depletion effect which dominates the formation of DNA nanoparticles. Our result reveals the possibility of promoting DNA nanoparticle formation by adjusting the solution environment. The result might be potentially useful in the gene therapy field where the formation of DNA nanoparticles is necessarily required.

## Methods

### Electrophoresis assay

Plasmid DNA pUC19 (2686 base pairs) was purchased from Bioron and stored in TE Buffer (10 mmol/l Tris–HCl, 1 mmol/l EDTA, pH 7.5). PEG 6K was purchased from Sigma (Germany). In order to maintain the bio-activity of DNA and HindIII, all the reaction mixtures contained 1X TE Buffer. If not specially mentioned, DNA cleavage mixtures were prepared by mixing 2 μL of plasmid DNA pUC19 solution (0.5 mg/ml), 2 μL of HindIII solution (10 unit/ml) and 2 μl of 10X Buffer R (1X Buffer R: 10 mM Tris-HCl pH = 8.5, 10 mM MgCl_2_ 100 mM KCl and 0.1 mg/ml BSA) and 14 μL of PEG 6K and TE solution to make the total volume up to 20 μL. The cleavage reactions were performed by incubating the reaction mixture at 37 °C for 1 h. The reactions were stopped by the addition of 2 μL of loading Buffer (0.05% bromophenol blue, 40% sucrose, 0.1 M EDTA (pH 8.0) and 0.5% SDS) into the 20 μL of mixtures. The entire 24 μL of the resulting solutions was loaded at once in a single gel hole to keep the amount of DNA the same in each lane, so that the brightness of DNA bands in the gel reflected both the concentration and the amount of DNA. Agarose gel (0.8%) containing 1 mg/ml ethidium bromide was used in the electrophoresis assays. The electrophoresis assay system and the gel imaging system with Image Lab^TM^ software were purchased from Biorad (USA).

### DLS measurement

We prepared the DLS samples with the same components as the reaction mixtures in electrophoresis assay. PEG 6K solutions were prepared with 1X TE Buffer so that all the samples contained 1X TE Buffer. In each sample we mixed 150 μl of pUC 19 (0.5 mg/ml), 750 μl of 50% (w/w) PEG 6K solution, and 600 μl of TE Buffer with or without Buffer R (or Mg^2+^). Thus, the concentrations of DNA, Buffer R and Mg^2+^ were the same as that in the electrophoresis assay. DLS measurement was carried out using a BI-200SM Goniometer at both 23 °C and 37 °C. The experimental results were fitted with the single component model described as below.

The characteristic decay time τ_D_ was obtained by evaluating the time-dependent autocorrelation function.


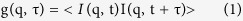


where *I*(q, t) is the intensity of light scattered from the samples at time *t* and at the wave vector *q.* The autocorrelation function *g*(*q*, τ) for single component mode is represented by,


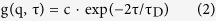


We fitted the DLS signal with eq 2 to obtain the value of τ_D_. Then we calculated the diffusion coefficient of the tracers D by


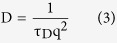


Here, *q* is the modulus of the scattering vector of the optical arrangement,


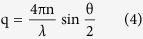


where *n* is the refractive index of the liquid solution measured by a standard Abbe refractometer (Carl-Zeiss, Germany), *λ* is the wavelength of the laser in vacuum and *θ* is the scattering angle. The signal intensity of DLS is proportional to the 6^th^ power of the size and the concentration of components. When the signal from one component overwhelmed the rest, we simply fitted the characteristic diffusion time *τ*_0_ of the overwhelming component with single-component model eq (2).

We obtained the size of the particles accoding to the Einstein-Stoke relation


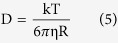


where *k* is Boltzmann’s constant, *T* is the temperature, *η* is the viscosity of the solution and R the hydrodynamic radius of the tracers. In a solution where the viscosity *η* is contant, τ_0_~R.

### FCS measurement

A commercially available NIKON EZ-C1 confocal microscope equipped with Pico Harp 300 FCS setup (PicoQuant, Germany) was used. A stable diode laser was used with a wavelength of 488 nm. We split the signal equally into two avalanche photodiode detectors and cross-correlated the FCS signal to eliminate after-pulsing. Each sample was measured three times and the three curves were averaged. All FCS measurements were performed at 37 °C.

66 bp dsDNA or 66 nt ssDNA labeled with ATTO 488 dyes at one end (Eurofins Germany) were used in the FCS measurement. The sequence of dsDNA and ssDNA is shown in the supporting information. The FCS signal follows a single-component model when no DNA aggregation occurs


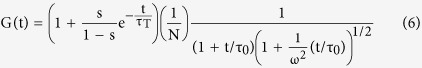


where s is the fraction of dye molecules in the triplet state, τ_T_ is the triplet lifetime, N is the average number of molecules in the focal volume, τ_0_ is the residence time of molecules in the focal volume (characteristic diffusion time), and ω is the structure parameter describing the ratio between the longitudinal and transverse size of the focal volume. The residence time τ_0_ is inversely proportional to the diffusion coefficient of tracers


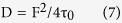


*F* is the size of focal volume in the x-y plane. We fixed the size of focal volume in this study, so the diffusion coefficient of the tracer *D* was inversely proportional to its residue time *τ*. According to eq 5, the size of the fluorescent DNA can be evaluated by the residence time τ_0_.

When t = 0


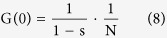


In this study we kept the triplet within 10% and roughly constant, and the amplitude G(0)~1/N. When we fixed the size of focal volume, G(0) was inversely proportional to the concentration of fluorescent DNA samples. The reflective index has a compound influence on the geometry of FCS focal volume. In the experiment, we carefully calibrated the experimental parameters to avoid the influence of PEG solution on the geometry of the focal volume. We immersed the focal volume into the PEG solution at a distance of 10 μm from the bottom interface so that the influence of PEG 6k on the geometry of the focal volume was minimized and insignificant (see the calibration in the supporting information).

### Analytical ultracentrifugation assay

The single-stranded DNA was purchased from Eurofins (Germany). The dsDNA labeled with FAM dye at both ends was produced by annealing complementary single-stranded DNA which was labeled with FAM dye at 5′ end. The concentration of dsDNA labeled with FAM was 20 nM in each sample. The sedimentation of DNA aggregates was performed using the Beckman-Coulter XL-I analytical ultracentrifuge equipped with an AU-FDS fluorescence detector system (AVIV Biomedical Inc.) The wavelength of the excitation light was 488 nm. The samples were loaded into two-sector charcoal-epon cells which have 12-mm pathlength and quartz windows. A low rotor speed of 5000 rpm was used. Data was collected at room temperature. The preparation of all samples took ca. 30 min. The measurements began after thermostatting and calibration.

### Fluorescence spectroscopy measurement

The LS 55 Spectrofluorimeter (Perkin Elmer U.K.) equipped with FL Winlab software was used to measure the fluorescence spectra at room temperature. A quartz cuvette with a 4 mm-path length for excitation and 10 mm for emission was used. The excitation wavelength was 488 nm. Fluorescein, the free FAM dye, was purchased from Sigma Aldrich Corp (Germany). The concentration of free FAM dye was 80 nM for each sample. 66bp dsDNA was labeled at both ends with FAM dye. The concentration of FAM-DNA was 40 nM for each sample.

## Additional Information

**How to cite this article**: Hou, S. *et al.* How can macromolecular crowding inhibit biological reactions? The enhanced formation of DNA nanoparticles. *Sci. Rep.* **6**, 22033; doi: 10.1038/srep22033 (2016).

## Supplementary Material

Supplementary Information
