# Supplementary material for: How can macromolecular crowding inhibit biological reactions? The enhanced formation of DNA nanoparticles
Source: Sci Rep. 2016 Feb 23;6:22033. doi: 10.1038/srep22033 (PMC4763241; doi:10.1038/srep22033)
Supplement: Supplementary Information [file srep22033-s1.pdf]

Supporting information for

How can macromolecular crowding inhibit biological reactions? The enhanced formation of DNA nanoparticles

Sen Hou[1], Piotr Trochimczyk[1], Lili Sun[1], Agnieszka Wisniewska[1], Tomasz Kalwarczyk[1], Xuzhu Zhang[1], Beata Wielgus-Kutrowska[2], Agnieszka Bzowska[2], Robert Holyst[1*]*

[1]Institute of Physical Chemistry PAS, Kasprzaka 44/52, 01-224 Warsaw, Poland

[2]Division of Biophysics, Institute of Experimental Physics, Faculty of Physics, University of Warsaw, al. Zwirki i Wigury 93, 02–089 Warsaw, Poland

Corresponding author: E-mail: hs0010910@gmail.com (Sen Hou); robert.holyst@gmail.com (Robert Holyst)

Table of Contents

S1 Influence of DNA type on their electrophoresis mobility

S2 Measurement of the reflective index of PEG 6k solution

S3 Density measurement for the solutions used in analytical ultracentrifugation assay

S4 Viscosity of PEG 6k solution in TE buffer measured by dynamic light scattering technique

S5 Viscosity of PEG 6k solution with Buffer R and Mg^{2+} measured by rheometer

S6 Calibration of FCS system: Influence of the position of the FCS focal volume on its size

S7 Calibration of FCS system: Influence of the position of the FCS focal volume on its shape

S8 Calibration of FCS system: Influence of macromolecular crowding on focal volume calibrated with Rhodamine 110 dyes

S9 Absorbance spectra of PEG 6k solution

S10 Study on the redistribution assay of PEG 6k in the analytical ultracentrifuge

S11 Influence of macromolecular crowding on BSA aggregation and BSA attachment to DNA

S12 DNA information

S13 Particles size measured in PEG 6k solution without DNA by DLS

S14 Discussion about the influence of viscosity

S15 Electrophoresis assay for DNA nanoparticles

1. Influence of DNA type on their electrophoresis mobility

Commercial available plasmid DNA pUC 19 has a super coiled structure which is composed of two complimentary single-stranded (ssDNA) DNA. Introducing cleavage into each ssDNA by restriction enzyme releases the topological force inside dsDNA. However the dsDNA is still of a circular structure which is stabilized by hydrogen bonds between complimentary ssDNA. Cleavage of both ssDNA strands at the close vicinity breaks the circular dsDNA into a linear one. The three types of dsDNA have different electrophoresis mobility. Cleavage of plasmid DNA by HindIII can be detected by their electrophoresis mobility (Fig S1).

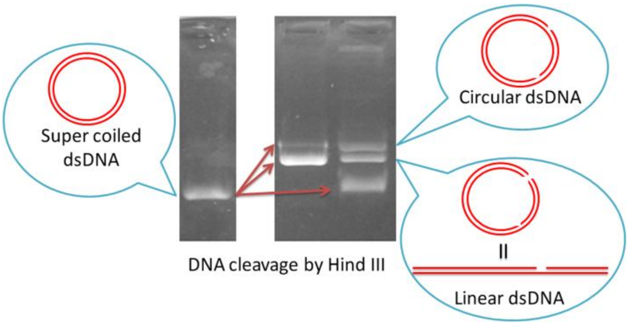

Fig. S1 The type of double-stranded (dsDNA) plasmid and their relative electrophoresis mobility.

2. Measurement of the reflective index of PEG 6k solution

The refractive index of PEG 6k solution was measured on a standard Abbe refractometer (Carl-Zeiss, Germany). The PEG 6k solutions were prepared with TE buffer. The influence of ions is assumed to be very small (Fig. S2). We used the measure of refractive index of PEG 6K in TE buffer for all kinds of the PEG 6k solutions including those containing Buffer R and Mg^{2+}.

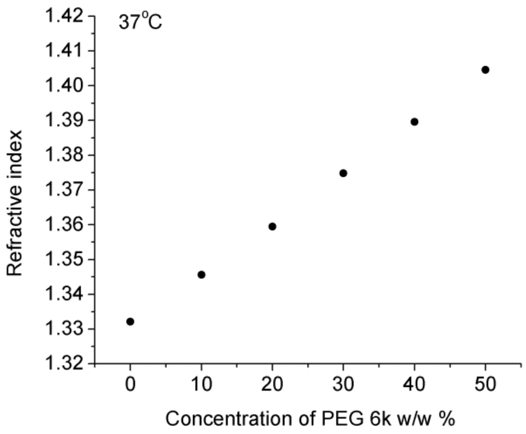

Fig. S2 Refractive index of PEG 6k solution at 37°C.

3. Density measurement for the solutions used in analytical ultracentrifugation assay

The density was calculated by the mass of solution divided by its volume. The density measurement was performed at 23°C which was the same as the temperature for analytical ultracentrifugation assay. All the measurements were repeated at least three times and the results were presented as the average value ± standard deviation (SD).

Table. S1 Density of relevant solutions for analytical ultracentrifuge assay

	Density (g/ml)
H_2O	1.002 ±0.005
H_2O+Buffer R	0.998 ±0.006
H_2O+$MgCl_2$	0.999 ±0.002
TE	1.002 ±0.002
TE+Buffer R	1.001 ±0.003
TE+$MgCl_2$	1.004 ±0.001
25% PEG 6k	1.039 ±0.003
25% PEG 6k+Buffer R	1.020 ±0.012
25% PEG 6k+$MgCl_2$	1.029 ±0.004

4. Viscosity of PEG 6k solution in TE buffer measured by dynamic light scattering technique

Viscosity of PEG 6k solutions prepared with TE was measured using our previous method employing a dynamic light scattering equipment.[1] A BI-200SM Goniometer (Brookhaven Instruments Corp.) equipped with an Argon Ion Stabilite 2017 laser was used. Nanobeads of R=44.04 nm in radius (Polysciences Inc.) were used as tracers for viscosity measurement. The solutions were filtered before measurement. Briefly we obtained the diffusion coefficient of the nanobeads D by DLS measurement. Then the viscosity, η, was calculated according to Stocks-Einstein equation $D = \frac{kT}{6\pi\eta R}$, where k is Boltzmann's constant and T is the temperature. Due to the huge size of nanobeads compared to PEG 6k (~2nm), the measured viscosity equals to the macroscopic viscosity. (see a discussion about nanoviscosity and macroviscosity in section S14).

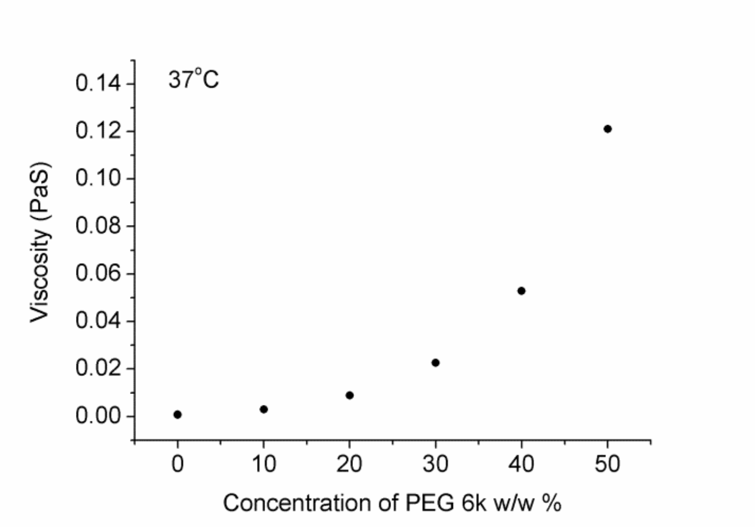

Fig. S3 Viscosity of PEG 6k solution with TE buffer at 37°C.

5. Viscosity of PEG 6k solution with Buffer R and Mg^{2+} measured by rheometer

The influence of Buffer R and Mg^{2+} on the viscosity of 25% PEG solutions were measured by the rotational rheometer Kinexus (by Malvern Instruments Ltd) in temperature 37°C. The controlled shear stress was in the range from 0.01 Pa to 1 Pa. The cone/plate geometry was used. The diameter of cone was 50 mm and angle was 1 degree. We used the flow curves (viscosity by shear rate) to calculate viscosity and extrapolated experimental points to zero. All the solutions were prepared with TE buffer. The concentration of Buffer R was the same as in the HindIII cleavage mixture. The concentration of Mg^{2+} was 10 mM which was the same as in Buffer R.

Table. S2 Influence of Buffer R and Mg^{2+} on the viscosity of PEG 6k solution. All the solutions were prepared with TE buffer. The data shows that the influence of both Buffer R and Mg^{2+} is negligible.

	Viscosity (mPaS)
25% (w/w) PEG 6k	14.27±0.08
25% (w/w) PEG 6k+Buffer R	17.00±0.16
25% (w/w) PEG 6k+Mg^{2+}	14.60±0.09

6. Calibration of FCS system: Influence of the position of the FCS focal volume on its size

We gradually put the position of FCS focal volume into the solution. Since the concentration of fluorescent dsDNA labeled with ATTO488 dyes was constant for the measurements on the same solution, the size of the FCS focal volume was probed by the number of dsDNA inside. The measurement with larger focal volume resulted in a large number of observed tracers inside. The initial zero position was calibrated as the position where the focal volume located on boarder of the solution. This position was detected as the appearance of a huge change in FCS original signal at the surface of liquid. The result shows the size of focal volume did not change obviously when the focal volume moved within a distance of 0-20 μm. In our study we set the distance to 10 μm, so that a small shift in the distance should not change the size of FCS focal volume.

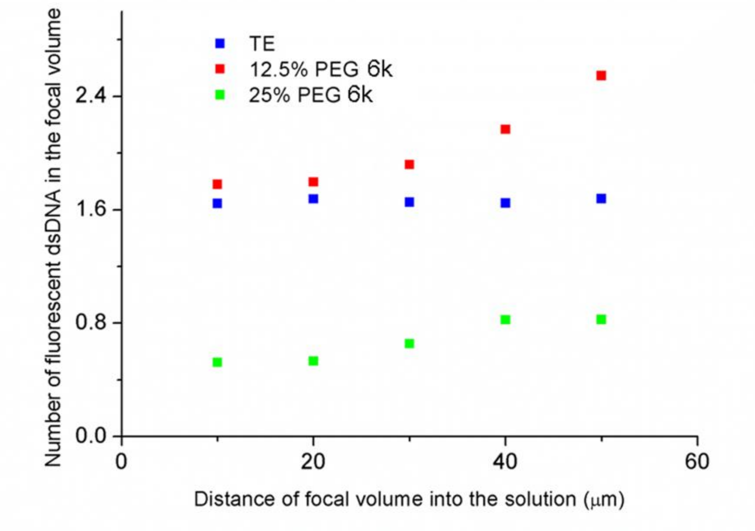

Fig. S4 Influence of the position of the FCS focal volume on the number of dsDNA inside. Within 20 μm, the number of DNA does not change obviously. This result shows the size of focal volume does not change obviously in this range. The concentration of dsDNA is different for different solutions.

7. Calibration of FCS system: Influence of the position of the FCS focal volume on its shape

When the size of focal volume is not changed, the shape of the focal volume can be monitored as the characteristic diffusion time of the tracer. We studied the characteristic diffusion time of dsDNA labeled with ATTO488 dye as a function of the position of the FCS focal volume. The result shows that the shape of focal volume did not change obviously when the focal volume moved within a distance of 0-20 μm. In our study, we set the position of the FCS focal volume at a distance of 10 μm, so that a small shift in the distance does not change the size of FCS focal volume.

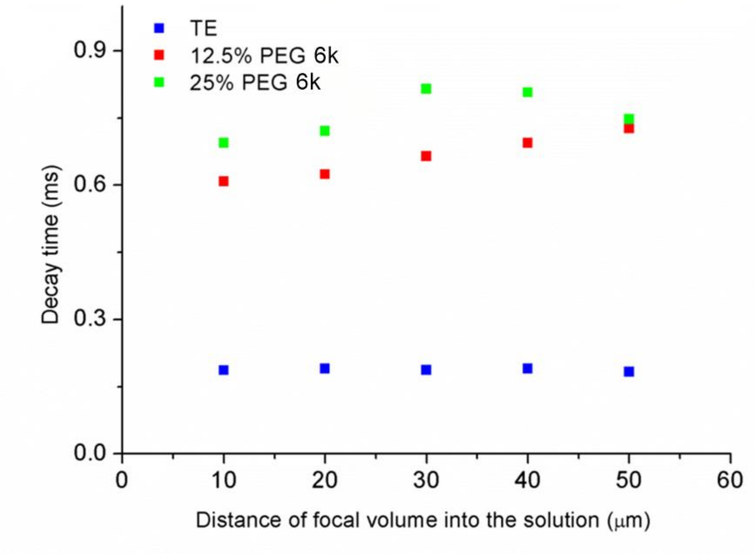

Fig. S5 Influence of the position of the FCS focal volume on the characteristic diffusion time of dsDNA labeled with ATTO488 dyes. This result shows the geometry of focal volume does not change when the focal volume enters the solutions within 20 μm. The concentration of dsDNA is different for different solutions.

8. Calibration of FCS system: Influence of macromolecular crowding on focal volume calibrated with Rhodamine 110 dyes

Rhodamine 110 was purchased from Sigma Aldrich Corp (Germany). A single-component model was fitted to the FCS curves to obtain the triplet proportion and G(0). The concentrations of Rhodamine were the same in all samples. All the solutions contained the same concentration of Buffer R as in HindIII cleavage system. The number of Rhodamine 110 in the focal volume were the same for TE, 12.5% and 25% PEG 6k solution. The result indicates that addition of PEG 6k should not change the size of focal volume (Fig. S6). Because of the different viscosity, the characteristic diffusion time differed for PEG 6k solutions of different concentrations.

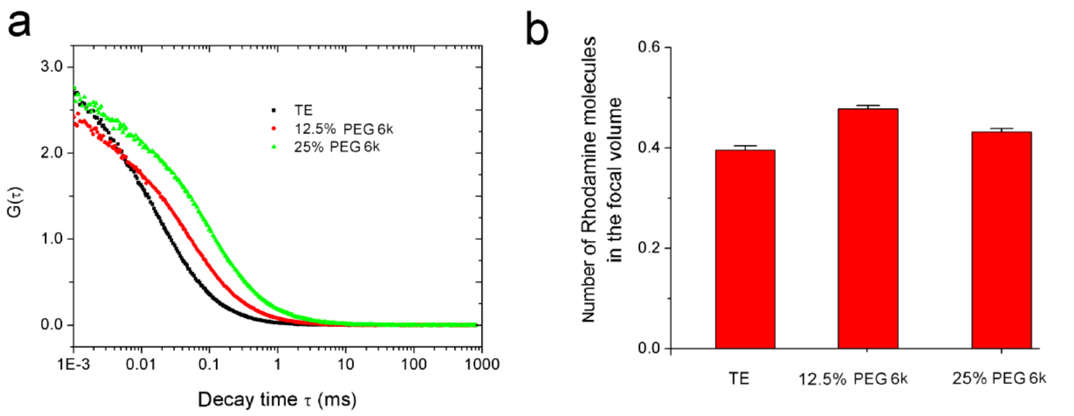

Fig. S6 FCS system calibrated with Rhodamine 110 dyes. a) FCS curves of Rhodamine in PEG 6k solutions. b) The number of Rhodamine molecules in the FCS focal volume. The FCS autocorrelation curves differ in their characteristic diffusion time but share a similar amplitude. The result indicates that the focal volume should not change significantly in PEG 6k solution of different concentrations.

9. Absorbance spectra of PEG 6k solution

The absorbance of PEG 6k solution was measured in a quartz curette with 10-mm optical pathlength, by using UV-visible spectrophotometer equipped with Thermo INSIGHT software (Thermo Scientific U.S.). 280 nm was used as the excitation wavelength for the measurement of the distribution of PEG 6k in the gravity field of the analytical ultracentrifuge assay.

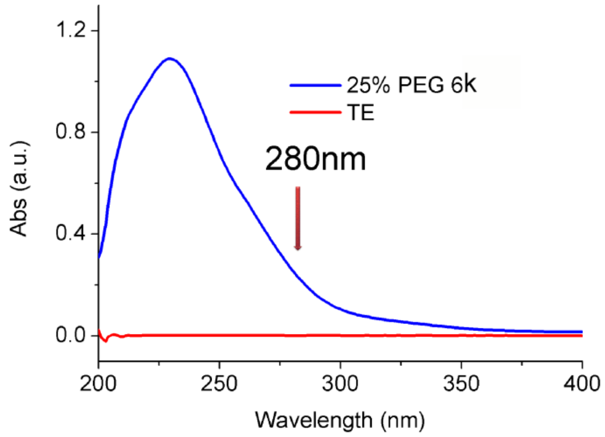

Fig. S7 Absorbance spectra of PEG 6k and TE buffer. 280 nm was used as the excitation wavelength for the measurement of the distribution of PEG 6k in the gravity field of the analytical ultracentrifuge assay

10. Study on the redistribution assay of PEG 6k in the analytical ultracentrifuge

The redistribution of PEG 6k in gravity field was recorded by analytical ultracentrifuge (Beckman Coulter U.S.) equipped with absorption optics. The observation (or absorption) wavelength was 280 nm. The samples were recorded every 10 min with a 50,000rpm rotation speed at room temperature. The PEG 6k was dissolved in TE buffer. The analytical ultracentrifugation essay was carried out by detecting the absorbance difference between the aimed sample and the blank sample. The samples were loaded into standard two-sector epon-charcoal cells which have 12-mm pathlength and quartz or sapphire windows. The positions of sample were located at 6.2~7.2 cm from the center of rotation. No redistribution was observed in the gravity field with a 50,000 rpm rotation speed in 150 min. (Fig S8)

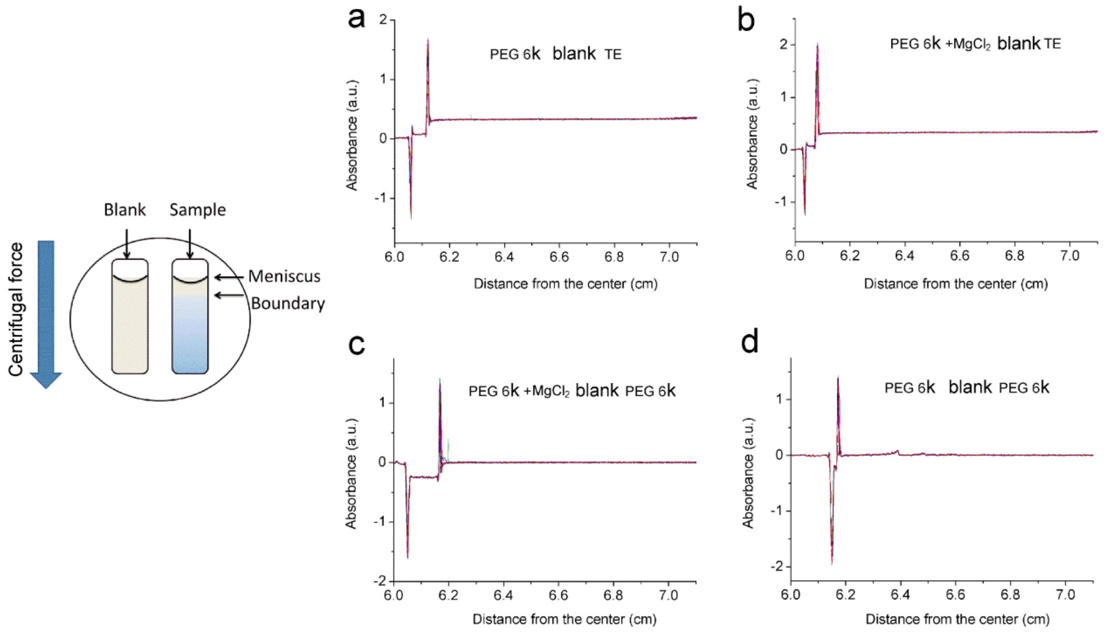

Fig. S8 PEG 6k does not redistributed under 50,000 rpm for 150 min in the analytical ultracentrifugation essay. a) We used PEG 6k as the sample and TE as the blank control. No redistribution of PEG 6k was observed. b) We used PEG 6k containing 10 mM Mg^{2+} as the sample and TE as the blank control. No redistribution of PEG 6k was observed. c) We used PEG 6k containing 10 mM Mg^{2+} as the sample and the PEG 6k solution of the same concentration as the blank control. Mg^{2+} did not cause a difference in the absorbance in PEG 6k solutions. d) The difference of the absorbance is 0 when the same PEG 6k solution was loaded as both sample and the blank control.

11. Influence of macromolecular crowding on BSA aggregation and BSA attachment to DNA

ATTO488 protein label kit were purchased from Sigma-Aldrich (Germany). We labeled BSA with ATTO 488 dye. The FCS signals were fitted and the numbers of ATTO488 labeled BSA in the focal volume were calculated in the same way as shown in the manuscript. The concentrations of ATTO488 labeled BSA were the same for all samples. All the solutions contained the same concentration of Buffer R as in HindIII cleavage system. After each measurement we added unlabeled plasmid DNA pUC 19 into the solution to see whether the macromolecular crowding of PEG 6k solution caused BSA molecules attached to DNA surface. Given BSA molecules attached to DNA, a larger characteristic decay time, (i. e. a slower diffusion of BSA) and a decreased number of BSA in the FCS focal volume (i. e. a decrease of BSA concentration) should be observed. However the result shows that macromolecular crowding should not induce attachment of BSA to DNA (Fig S9). In the same way, we conclude that no BSA aggregation occurs in PEG 6k solution. In our previous work, we show the HindIII enzymes are not denaturated in PEG 6k solution.[1]

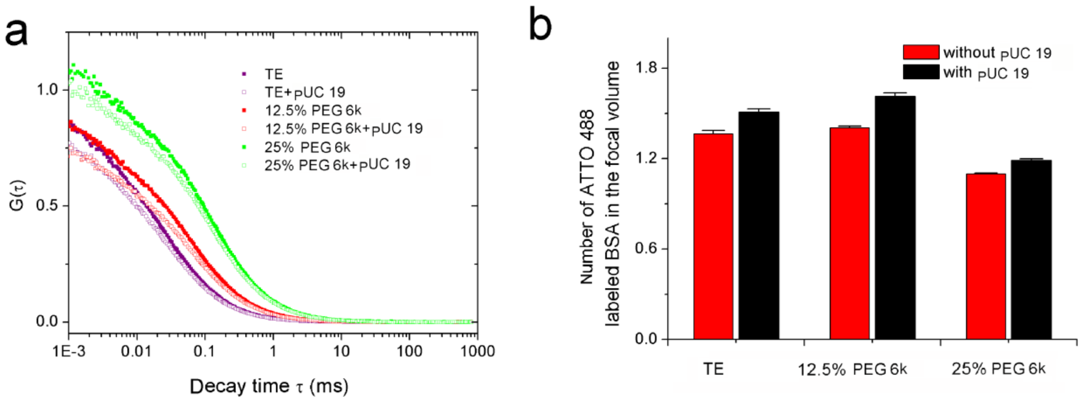

Fig. S9 We labeled BSA with ATTO 488 dyes. Neither macromolecular crowding of PEG 6k nor addition of extra DNA (unlabeled plasmid DNA pUC 19) had an obvious effect on the a) FCS autocorrelation curves and b) the number of BSA in the focal volume. The result indicates that: 1) Macromolecular crowding of PEG 6k should not induce aggregation of BSA molecules; 2) Macromolecular crowding of PEG 6k should not induce BSA molecules attached to DNA.

12. DNA information

dsDNA labeled with ATTO550 dye at one end

ATTO488-5’-GATACGAGCGCAAAGCCCGTAGGTATTGGAAAGCTTTCCCTATTCTCG GTCGAATTCATTAGCCAT-3’

3’-CTATGCTCGCGTTTCGGGCATCCATAACCTTTCGAAAGGGATAAGAGCCAGCTT AAGTAATCGGTA-5’

ssDNA labeled with ATTO550 dye

ATTO488-5’-GATACGAGCGCAAAGCCCGTAGGTATTGGAAAGCTTTCCCTATTCTCG GTCGAATTCATTAGCCAT-3’

dsDNA labeled with FAM dye at both ends

FAM-5’-GATACGAGCGCAAAGCCCGTAGGTATTGGAAAGCTTTCCCTATTCTCGGTC GAATTCATTAGCCAT-3’

3-CTATGCTCGCGTTTCGGGCATCCATAACCTTTCGAAAGGGATAAGAGCCAGCTTA AGTAATCGGTA-5-FAM

13. Particles size measured in PEG 6k solution without DNA by DLS

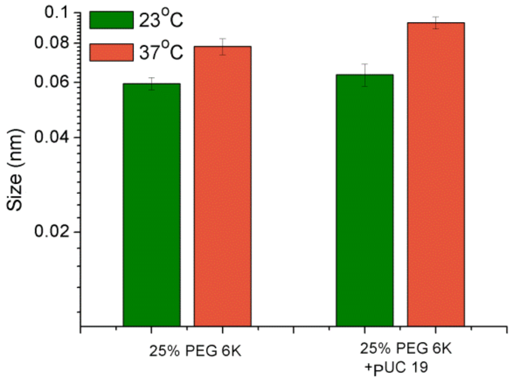

Fig S10. The size of particles in PEG 6k solution with/without DNA measured by DLS.

The signal intensity of DLS is proportional to the 6th power of the size and the concentration of components. The signal from plasmid DNA was overwhelmed by the signal from PEG 6k, thus the information of plasmid DNA was not detected in 25% PEG 6k solution.

The particle size in the PEG 6k solution without Buffer R is measured to be smaller than 1 nm. However this measure does not contradict with the size of PEG 6k ~ 2nm. Actually what we measure in PEG 6k solution is the value of "blob" size (the correlation length of PEG 6k network).[2] In PEG 6k solution, the polymers form an overlapping network. The "blob" size ξ is defined as the size that all monomers in a polymer belong to the same polymer chain. It is a function of polymer concentration, x, $\xi = R_{\mathrm{g}}(\mathrm{x}/\mathrm{x}^{*})^{-0.75}$. Here x* is the concentration at which the polymer chains start to overlap. It is defined as $\mathrm{x}^{*} = M_{p}/(4/3\pi R_{\mathrm{g}}^{3} N_{\mathrm{A}})$. Mp is the molar mass of the polymer and N_A is Avogadro's number. For PEG 6k x* = 0.08 g cm^{-3}. Given the size of PEG 6k is ~2 nm according to its radius of gyration,[1] the "blob" size of 25% PEG 6k solution should be ~0.9 nm. These data agrees with the "blob" size of PEG 6k solution measured by DLS.

14. Discussion about the influence of viscosity

An apparent influence of macromolecular crowding is the increased viscosity. It slows down the diffusion of the enzymes and the DNA substrates and herein reduces the reaction rate. In a reaction described as $A + B \leftrightarrow AB$, if the limiting step is the encounter of A with B, macromolecular crowding can decrease the reaction rate by reducing diffusion.[3] However the real viscosity experienced by the molecules in the macromolecular crowding environment (defined by our early work as nanoviscosity[1,2,4]) is always not too high. The nanoviscosity is different from the macroviscosity measured with a falling ball test.[5,6,7,8] The nanoviscosity η_{nano} experienced by a probe with a hydrodynamic radius r in a macromolecular crowding solution composed of polymers with a hydrodynamic radius R is a function of the size of both the polymer and the probe $\eta_{\text{nano}} = \eta_0 \exp\,[\left(\frac{Rr}{\sqrt{R^2+r^2}\,\xi}\right)^a]$.[4] Here η_0 is the viscosity of water, ξ is the correlation length interpreted as the average distance between the points of the entanglement in the polymer network, a is a constant of order 1 and slightly differs from system to system. When the size of probe is much larger than the size of polymer r>>R, the experienced viscosity derived from the above function takes the form of macroviscosity as $\eta_{\text{macro}} \approx \eta_0 \exp\,[\left(\frac{R}{\xi}\right)^a]$. Herein we get the relation between nanoviscosity and macroviscosity as $\eta_{\text{nano}}/\eta_{\text{macro}} = \exp\,[\left(\frac{R}{\sqrt{(R/r)^2+1}}\frac{1}{\xi}\right)^a - \left(\frac{R}{\xi}\right)^a]$. It is obvious that the nanoviscosity experienced by a nanoscale probe η_{nano} is always smaller than the macroscopic viscosity of the solution η_{macro}. In our study we use PEG 6k as the macromolecules. The macroviscosity measured by rheometer of 25% PEG 6k solution with or without Buffer R is merely about 15 times larger than the viscosity of water. Given the nanoviscosity experienced by DNA and enzymes is smaller than the macroviscosity, the change of nanoviscosity by adding PEG 6k is estimated to be within 1 order of magnitude. Consequently although the diffusion of DNA and enzyme may be slightly decreased in PEG 6k solution, the viscosity should contribute insignificantly to the complete halt of the DNA cleavage.

15. Electrophoresis assay for DNA nanoparticles

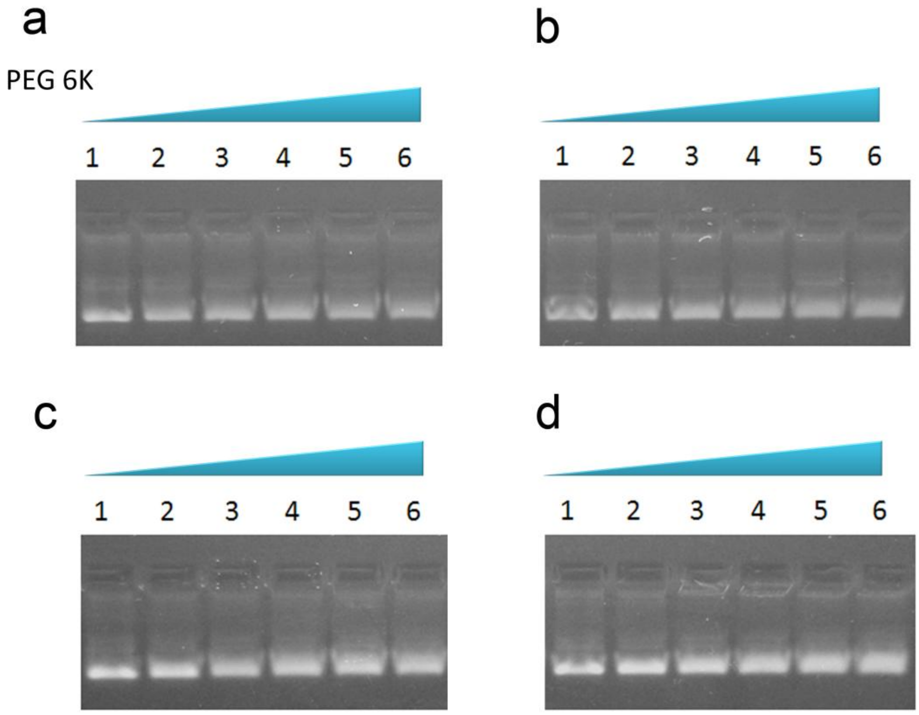

Fig. S11 Formation of DNA nanoparticles in PEG 6k solution cannot be observed by electrophoresis assay. The concentrations of PEG 6k are 0%, 6.25%, 12.5%, 18.75%, 25% and 31.25% respectively. To mimic the HindIII cleavage system, we supplemented each sample with Buffer R of the same concentration as in the cleavage system. No HindIII was added.

DLS signal shows the formation of large DNA nanoparticles in PEG 6k; however, the formation of DNA nanoparticles cannot be observed as their decreased mobility in electrophoresis assay (Fig. S11). During electrophoresis assay, the macromolecular crowding solution is gradually replaced by the electrophoresis buffer. Therefore the DNA nanoparticles disassembles in the agarose gel.

References

1. Hou, S., *et al.* Influence of nano-viscosity and depletion interactions on cleavage of DNA by enzymes in glycerol and poly(ethylene glycol) solutions: qualitative analysis. *Soft Matter* **7**, 3092-3099 (2011).
2. Holyst, R., *et al.* Scaling form of viscosity at all length-scales in poly(ethylene glycol) solutions studied by fluorescence correlation spectroscopy and capillary electrophoresis. *Phys. Chem. Chem. Phys.* **11**, 9025-9032 (2009).
3. Ellis, R. J. Macromolecular crowding: obvious but underappreciated. *Trends Biochem. Sci.* **26**, 597-604 (2001).

4. Kalwarczyk, T., *et al.* Comparative Analysis of Viscosity of Complex Liquids and Cytoplasm of Mammalian Cells at the Nanoscale. *Nano Lett.* **11**, 2157-2163 (2011).
5. Lin, T. H. & Phillies, G. D. J. Probe Diffusion in Poly(Acrylic Acid) Water - Effect of Probe Size. *Macromolecules* **17**, 1686-1691 (1984).
6. Jena, S. S. & Bloomfield, V. A. Probe diffusion in concentrated polyelectrolyte solutions: Effect of background interactions on competition between electrostatic and viscous forces. *Macromolecules* **38**, 10551-10556 (2005).
7. Wattenbarger, M. R., Bloomfield, V. A., Bu, Z. & Russo, P. S. Tracer Diffusion of Proteins in DNA Solutions. *Macromolecules* **25**, 5263-5265 (1992).
8. Busch, N. A., Kim, T. & Bloomfield, V. A. Tracer diffusion of proteins in DNA solutions. 2. Green fluorescent protein in crowded DNA solutions. *Macromolecules* **33**, 5932-5937 (2000).
